# Interface coupling in Au-supported MoS_2_–WS_2_ heterobilayers grown by pulsed laser deposition†

**DOI:** 10.1039/d3nr00614j

**Published:** 2023-03-31

**Authors:** Paolo D'Agosta, Francesco Tumino, Valeria Russo, Andrea Li Bassi, Carlo S. Casari

**Affiliations:** a Department of Energy, Politecnico di Milano via G. Ponzio 34/3 I-20133 Milan Italy francesco.tumino@queensu.ca paolo.dagosta@polimi.it; b Department of Physics, Engineering Physics and Astronomy, Queen’s University 64 Bader Lane Kingston ON Canada K7L 3N6

## Abstract

Van der Waals heterostructures of transition metal dichalcogenides (TMDs) are promising systems for engineering functional layered 2D materials with tailored properties. In this work, we study the growth of WS_2_/MoS_2_ and MoS_2_/WS_2_ heterobilayers by pulsed laser deposition (PLD) under ultra-high vacuum conditions. Using Au(111) as growth substrate, we investigated the heterobilayer morphology and structure at the nanoscale by *in situ* scanning tunneling microscopy. Our experiments show that the heterostructure growth can be controlled with high coverage and thickness sensitivity by tuning the number of laser pulses in the PLD process. Raman spectroscopy complemented our investigation, revealing the effect of the interaction with the metallic substrate on the TMD vibrational properties and a strong interlayer coupling between the MoS_2_ and WS_2_ layers. The transfer of the heterobilayers on a silica substrate *via* a wet etching process shows the possibility to decouple them from the native metallic substrate and confirms that the interlayer coupling is not substrate-dependent. This work highlights the potential of the PLD technique as a method to grow TMD heterostructures, opening to new perspectives in the synthesis of complex 2D layered materials.

## Abstract 2

Epitaxial growth of MoS_2_–WS_2_ heterobilayers monitored by scanning tunneling microscopy with insight into interface effects provided by Raman spectroscopy.

## Graphical abstract

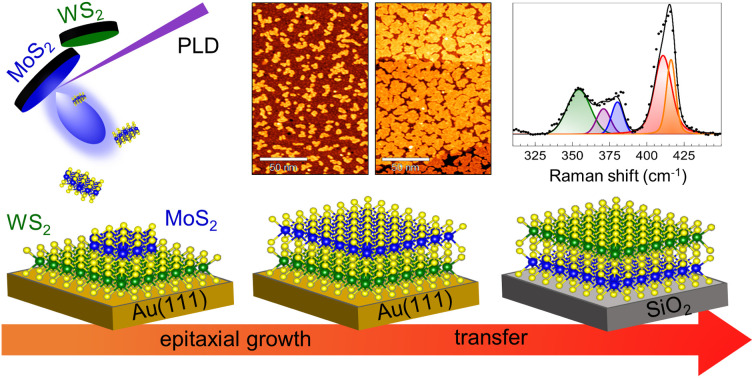

## 1 Introduction

The transition metal dichalcogenide (TMD) class has risen to a prominent position in the research field of 2D materials, as it includes many layered solids that can be reduced to single layers (SL) with intriguing optical and electronic properties.^1^ The wide variety of physical characteristics shown by TMDs can be further enriched by combining different TMD layers into vertically stacked van der Waals (vdW) heterostructures. WS_2_/MoS_2_ heterobilayers are the prototypical TMD vdW heterostructure, formed by stacking SL WS_2_ onto SL MoS_2_. This heterostructure is a type II semiconducting junction (*i.e.* staggered gap heterojunction) with promising optoelectronic^2,3^ and photocatalytic^4^ properties.

The fabrication of TMD heterobilayers is still a challenging and crucial task for future applications in real devices. Since stacking single layers through the exfoliation procedure requires micrometer precision and is difficult to scale up, the ideal fabrication method should be based on a bottom-up synthesis approach that offers highly precise control over thickness, uniformity over a relatively large area, good crystal quality, and purity of the interface between different layers. To assess suitable synthesis routes, it is thus important to investigate the heterostructure growth *in situ* and to gain information on several significant factors, such as the interaction with the growth substrate, the formation of defects, and the potential alloying between the heterostructure components. The most common fabrication approaches rely on transferring and stacking previously prepared single-layer flakes^5^ or on direct growth by chemical vapor deposition (CVD).^6^ However, alternative physical vapor deposition (PVD) methods have shown the possibility to produce high-quality TMD layers and heterostructures with high control over the growth process under low contamination conditions.^7–10^

Among PVD techniques, pulsed laser deposition (PLD) has been recently used for the synthesis of MoS_2_ and WS_2_ layers over relatively large (∼cm^2^) areas.^11–16^ This technique is based on target ablation by intense laser pulses, which produce a plasma plume consisting of highly energetic species that condense on the substrate. The growth process approximately retains the target stoichiometry and results in high deposition fluxes and large nucleation densities. Such characteristics make PLD suitable to grow complex multi-elemental films, albeit applications in the field of 2D heterostructures are still limited.^9^

In addition to the synthesis technique, the growth substrate can influence the properties of TMD layers and heterostructures. For instance, gold surfaces have been used as substrates for single-layer MoS_2_, favoring the formation of large-area flakes.^11,17–19^ However, the interaction with metals affects the electronic and vibrational properties of TMD layers (*e.g.* through induced strain and doping, or charge transfer phenomena),^12,20,21^ with important consequences for the design of efficient contacts in electronic devices.

In this work, we synthesized WS_2_/MoS_2_ and the inverse heterostructure MoS_2_/WS_2_ on Au(111) by PLD under ultra-high vacuum (UHV) conditions. This experimental approach allowed us to investigate the heterostructure growth *in situ* and to study its morphology and structure at the nanoscale by scanning tunneling microscopy (STM). Subsequently, we managed to transfer both heterobilayers on a silica support. Raman spectroscopy performed before and after the transfer revealed how the interaction with the gold substrate influences the vibrational properties of the base TMD layer, *i.e.* at the interface with the gold surface. Moreover, it proved that it is possible to transfer PLD-grown heterobilayers without weakening the strong interlayer coupling that originates from a fabrication process under UHV conditions. Our work explores the capability of the PLD technique to produce TMD heterobilayers with high coverage and thickness control, and provides insight into vibrational effects related to the interface with the growth substrate and the coupling between the TMD layers.

## 2 Materials and methods

All experiments were performed in a UHV system (base pressure in the 10^−10^ mbar range) composed of a chamber for STM measurements interconnected with a dedicated chamber for PLD. Au(111)/mica substrates (MaTeck) were cleaned by ionized Argon sputtering and annealing until the surface showed no contaminants and wide terraces. PLD was performed using stoichiometric MoS_2_ and WS_2_ targets (Testbourne) mounted on a rotating target holder. A KrF laser (248 nm, 20 ns pulse duration) was used to ablate the targets with a pulse energy of 200 mJ and a fluence of 2 J cm^−2^. Depositions were performed at a rate of 1 pulse per second to easily count the number of laser pulses on the target and finely tune the amount of deposited material. During depositions, the substrate was kept at room temperature (RT), 3 cm away from the target. After every PLD cycle, the sample was annealed at 750 K for 30 min in UHV to favor the crystallization of the deposited material. STM measurements were carried out at RT with an Omicron microscope using electrochemically etched tungsten tips. STM images were analyzed using Gwyddion.^22^ The top layer coverage was estimated by measuring the projected area of top layer islands and is expressed in fractions of monolayer (ML), where 1 ML corresponds to 100% projected area. Raman spectroscopy was performed *ex situ* with an InVia Renishaw spectrometer coupled to an Ar laser. All spectra were acquired using a 457 nm excitation wavelength focused by a 50× objective, with 1 mW of laser power on the sample. Raman peaks were fitted using Voigt functions after background subtraction. The Au-supported samples were then transferred on 300 nm thick SiO_2_ films thermally grown on Si. The transfer was achieved *via* the following wet etching procedure (see also ESI, Fig. S1†). First, the sample was set afloat on a 37% (w/w) HCl solution to help peel the Au film off from the mica substrate. Then, it was placed on the silica surface with the Au film facing up, so to have the TMD heterobilayer sandwiched between SiO_2_ and Au. Finally, gold was etched off using a potassium monoiodide solution. The remaining sample was cleansed in deionized water and isopropanol.

## 3 Results and discussion

**Fig. 1:**
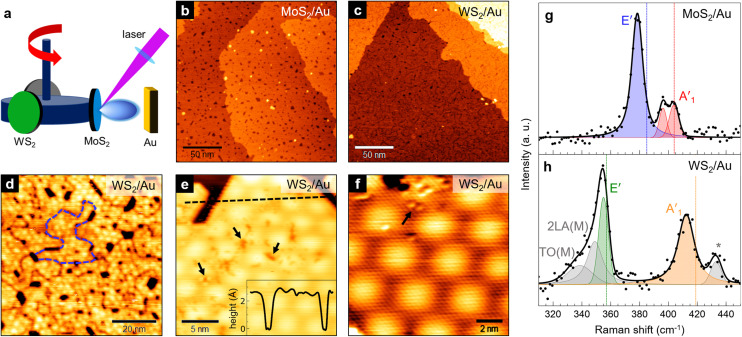
(a) Schematic representation of the PLD setup, where targets are mounted on a rotating carousel that allows to choose the material to be deposited. (b) and (c) Large-scale STM images of a single layer of (b) MoS_2_ on Au(111) (1.1 V, 0.4 nA) and (c) WS_2_ on Au(111) (1.3 V, 0.3 nA). (d) 100 × 100 nm^2^ STM image of the WS_2_/Au(111) surface, where grain boundaries between nanocrystals are visible (see *e.g.* the dashed blue line) (1.4 V, 0.3 nA). (e) Small-scale STM image of WS_2_/Au(111) showing surface defects and the hexagonal moiré pattern (1 V, 0.4 nA). Inset: topographic profile along the dashed black line. (f) Atomic resolution STM image of WS_2_/Au(111) (−0.27 V, 0.4 nA). Black arrows in (e) and (f) indicate point defects on the WS_2_ surface. (g) and (h) Raman spectra (at 457 nm) of our PLD-grown MoS_2_/Au(111) (g) and WS_2_/Au(111) (h). Legend: black dots = experimental points; solid black line = total fit; colored curves = labeled Voigt functions contributing to the fit. Vertical dotted lines in (g) and (h) mark the reference peak positions for exfoliated single layers as reported in ref. 23–25.

The growth of MoS_2_–WS_2_ heterobilayers was achieved using a sequential approach: first, we grew the bottom layer on Au(111); then, after selecting the second target for PLD, we deposited the top layer (Fig. 1a). With this procedure, we could synthesize two different heterobilayer architectures: one with MoS_2_ as the bottom layer and WS_2_ on top (denoted as WS_2_/MoS_2_/Au), and the inverse architecture with WS_2_ at the bottom and MoS_2_ on top (*i.e.* MoS_2_/WS_2_/Au).

Before considering the growth of the heterobilayers, we first discuss the main features of single-layer (SL) MoS_2_ and WS_2_ on Au(111) grown by PLD, which were used as base layers for the heterostructures. SL MoS_2_ on Au(111) can be grown by PLD with a proper number of laser pulses on the target, followed by thermal treatment at 750 K for 30 min. We described in detail this preparation procedure in a previous paper,^11^ where we showed that by increasing the number of pulses (typically in the 10–20 range) MoS_2_ nanocrystals form on the Au surface and gradually coalesce into a continuous film. The STM image in Fig. 1b shows the typical surface morphology of such a film, grown with a single deposition cycle of 11 laser pulses. Apart from small pits of uncovered substrate, originating from the coalescence of differently oriented nanocrystals that grow epitaxially along specific crystallographic directions (see ESI, Fig. S2a†), the MoS_2_ layer covers uniformly the Au surface and only a negligible amount of second layer clusters can be observed. The achieved MoS_2_ coverage is 0.92 ML; the bottom layer follows a layer-by-layer growth model until a value of around 0.9 ML is reached, after which second-layer islands start to nucleate where the film is locally complete. This poses some limitations in obtaining a full 1 ML coverage through PLD without depositing a non-negligible amount of second-layer islands.

We applied the same method to grow WS_2_ on Au(111) through a single deposition cycle of 13 laser pulses, at the same deposition and annealing parameters (for details, see Materials and methods). We obtained a single WS_2_ layer with a surface morphology very similar to that of MoS_2_ (Fig. 1c and d). The layer height measured by STM with respect to the Au surface is 2.4 ± 0.2 Å (inset of Fig. 1e), compatible with that of SL MoS_2_ (2.4 ± 0.1 Å,^11,26^ as shown in the ESI, Fig. S2c†). At high resolution (Fig. 1f), small ordered regions can be found showing an hexagonal moiré pattern with a 32 ± 1 Å periodicity and a lattice parameter of 3.20 ± 0.15 Å. Within uncertainty limits, these values are compatible to those reported for MoS_2_/Au(111) – respectively 33 ± 1 Å and 3.18 ± 0.12 Å (ref. 11, 17 and 27) (see also ESI, Fig. S2b†). Indeed, both TMD materials have the same atomic structure and similar lattice constants (the bulk value of the lattice parameter is 3.15 Å for both MoS_2_ ^28^ and WS_2_ ^29^). Defects are frequently observed on the surface of MoS_2_/Au and WS_2_/Au, both in the form of point defects (black arrows in Fig. 1e and f), which can be attributed to sulfur vacancies,^30^ and line defects due to grain boundaries between differently oriented nanocrystals^26^ (visible in Fig. 1d).

The Au-supported MoS_2_ and WS_2_ single layers shown above were characterized by Raman spectroscopy to study their vibrational properties. Fig. 1e and f report the Raman spectra of MoS_2_/Au (e) and WS_2_/Au (f) in the region of the two main Raman active modes, namely E′ and A′_1_, respectively corresponding to the in-plane and out-of-plane vibrations. Remarkable differences can be noted in comparison to the Raman literature of SL MoS_2_ and WS_2_ exfoliated onto inert substrates,^23^ which are reported as dotted vertical lines. Typically, in exfoliated SL MoS_2_ E′ is at 385 cm^−1^ and A′_1_ at 404 cm^−1^,^24,31^ whereas in exfoliated SL WS_2_ E′ and A′_1_ are at 357 and 419 cm^−1^, respectively.^25^

In our MoS_2_/Au(111) (Fig. 1g), E′ downshifts to 378 cm^−1^ (blue curve), while the out-of-plane mode can be deconvolved into two peaks (red curves) at 396 and 403 cm^−1^, respectively. Such splitting has been previously observed^32,33^ and can be attributed to the symmetry breaking along the out-of-plane direction due to a relatively strong interaction with the substrate. The latter is also responsible for the significant damping of the peak intensity compared to E′, with respect to the exfoliated SL (here and in the following, peak intensity refers to maximum peak height). The downshift of both in-plane and out-of-plane vibrations with respect to literature values can be attributed to strain and doping effects induced by the epitaxy on Au(111),^21^ affecting both peaks to different extents. Hence, as we previously reported,^11^ we measure a frequency difference between A′_1_ and E′ of 25 cm^−1^, *i.e.* larger than that expected for exfoliated SL MoS_2_ (19 cm^−1^); for the position of the A′_1_ peak, we considered the more upshifted contribution, which is closer in position to the A′_1_ peak in exfoliated SL MoS_2_.

The interaction with Au also influences the vibrational properties of SL WS_2_. In the spectral region shown in Fig. 1h, the low-frequency feature can be deconvolved into three contributions: a major peak (green curve) at 355 cm^−1^ attributed to E′, and two broader and less intense peaks (grey curves) at 349 and 338 cm^−1^, which can be tentatively assigned to second-order longitudinal acoustic and in-plane transverse optical modes^34^ – respectively labeled as 2LA(M) and TO(M) – whose contributions in Raman spectra are enhanced by defects. In the high-frequency region, the main feature (orange curve) at 412 cm^−1^ is associated to the out-of-plane A′_1_ mode, while the origin of the secondary feature (grey curve) at 433 cm^−1^ is currently unknown. Given the large frequency difference (21 cm^−1^) and the unequal peak intensities, we exclude that the origin of the latter mode is related to the same substrate-induced splitting that we observe in SL MoS_2_. It might instead be associated to the A_2u_ mode, which in exfoliated SL WS_2_ is at 441 cm^−1^ and only partially Raman-active for excitation wavelengths close to the resonance with the C exciton state (443 nm).^35^ As discussed above for MoS_2_/Au, both E′ and A′_1_ modes are downshifted due to the interaction with Au, resulting however in a smaller A′_1_–E′ frequency difference (57 cm^−1^) when compared to exfoliated SL WS_2_ (62 cm^−1^). Indeed, the frequency of the E′ mode is less sensitive to the influence of Au in SL WS_2_ than in SL MoS_2_.

The single MoS_2_ and WS_2_ layers grown on Au(111) are the bases for the growth of WS_2_/MoS_2_/Au and MoS_2_/WS_2_/Au heterostructures, respectively. Since several factors can influence the amount of material deposited from the target, including laser energy, pulse repetition rate and target-sample distance, we decided to keep such parameters constant and only investigate the effect of the cumulative number of laser pulses on the morphology and thickness of the resulting layer. In the following, we will discuss in detail the growth of WS_2_/MoS_2_/Au through its monitoring by STM for an increasing number of laser pulses on the WS_2_ target. The morphology evolves from isolated nanocrystals to a continuous film and, by increasing the pulse number beyond what is shown in this paper, to multilayer configurations.

**Fig. 2:**
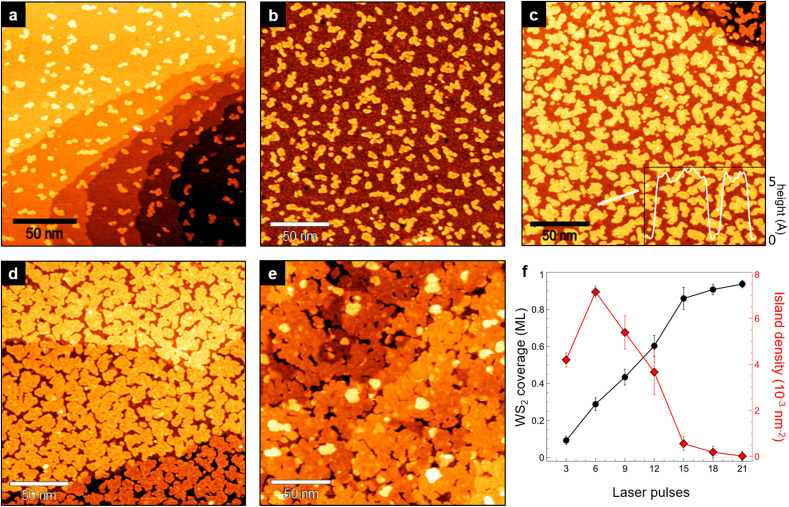
(a)–(e) Large-scale STM images of WS_2_ on MoS_2_/Au(111) observed after the 1^st^, 2^nd^, 4^th^, 5^th^ and 7^th^ growth cycle (a single cycle consists of PLD with 3 laser pulses and annealing at 750 K for 30 min). The WS_2_ coverage is measured after each cycle and expressed in fractions of monolayer (ML). (a) 0.09 ML (1.5 V, 0.4 nA). (b) 0.29 ML (−0.5 V, 0.3 nA). (c) 0.6 ML (1.2 V, 0.6 nA) (inset: topographic profile along the white line). (d) 0.86 ML (0.8 V, 0.7 nA). (e) 0.94 ML, with 0.05 ML of second-layer WS_2_ islands (1.35 V, 0.3 nA). (f) WS_2_ coverage (black dots) and island density (red squares) as a function of the number of laser pulses. Error was estimated by averaging several values of coverage and island density in different regions of the samples.

To monitor the growth of WS_2_ on the MoS_2_/Au layer, we applied a sequential preparation procedure in which a single growth cycle consists of PLD (with 3 laser pulses) and post-deposition annealing at 750 K. After each cycle, we performed STM measurements to monitor the growth and characterize the sample morphology (Fig. 2). The first 3 pulses (Fig. 2a) cause the formation of small irregular WS_2_ islands randomly distributed on the MoS_2_/Au surface. These islands grow larger after the second and third cycles (Fig. 2b and c) until they start to coalesce forming a continuous layer that covers almost completely the underlying MoS_2_ (Fig. 2d and e). The apparent height of WS_2_ islands measured with respect to the MoS_2_ surface is 5.5 ± 0.5 Å (inset of Fig. 2c), which remains consistent at different bias voltages in the −1.5 V to 1.5 V range. This value is lower than the interlayer distance in both bulk materials (6.15 Å)^28,29^ and compatible with that of WS_2_/MoS_2_ heterostructures grown *via* molecular beam epitaxy (MBE).^10^ The WS_2_ coverage increases linearly with the number of laser pulses (Fig. 2f, black dots), while the island density increases in the first two cycles and then gradually decreases due to coalescence (Fig. 2f, red squares). As we discussed for the bottom layer, we only observe second-layer WS_2_ islands after a coverage above 0.9 ML is reached (Fig. 2e). This indicates that the heterobilayer growth follows the layer-by-layer model at the initial stages of the process, but later becomes more complex as the coverage approaches layer completion, and that it is not possible to completely fill the holes generated by nanoisland coalescence without depositing a non-negligible fraction of second-layer WS_2_.

**Fig. 3:**
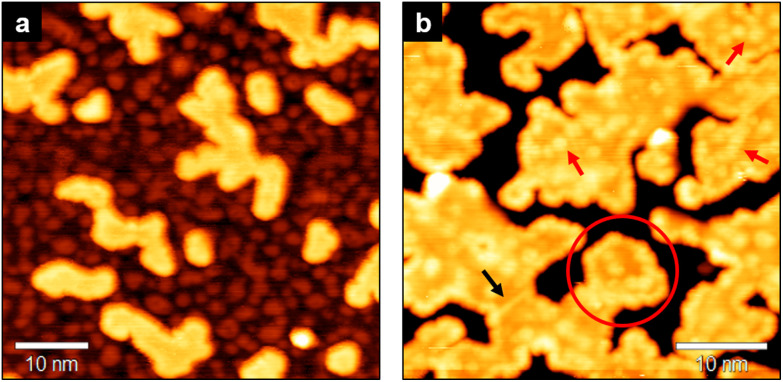
Small-scale STM images of WS_2_ on MoS_2_/Au(111), showing morphological details at different WS_2_ coverage. (a) Disordered surface corrugation observed on MoS_2_ layer, induced by the growth of WS_2_ islands (−1.6 V, 0.3 nA). (b) Surface morphology of the WS_2_ layer (1.1 V, 0.4 nA). The black arrow points to a grain boundary between adjacent nanocrystals, while the red arrows indicate some point defects in the WS_2_ islands, appearing as bright spots. The red line encircles an area where straight WS_2_ edges are observed.

As observed in higher resolution STM images (Fig. 3), the nucleation and growth of WS_2_ islands are accompanied by a larger disorder in the surface morphology of the underlying layer, where the moiré pattern is replaced by a more irregular modulation (Fig. 3a). This effect might be due to the PLD process, which involves the generation of a plasma plume that in vacuum can reach thousands of kelvins and that includes neutral and ionic species from the target. During deposition, such species impinge on the sample at high energy, and likely increase the amount of surface defects in the base layer. A second possibility is that the interference effect generating the moiré pattern is in some way modified by the presence of irregular WS_2_ nanoislands on top of the MoS_2_ bottom layer. Indeed, the emergence of the moiré pattern is a combination of topographical and electronic modulation effects,^36^ which may be influenced by the top-layer irregular islands. Since this effect is visible also when growing homobilayer MoS_2_ (see Fig. S3 of the ESI†), we exclude large Mo–W intermixing as a possible cause, although a low amount of intermixing promoted by the annealing treatments cannot be ruled out. The irregular shape of WS_2_ islands is likely due to limited mobility on the MoS_2_ surface,^37^ possibly hindered by MoS_2_ defects even at high annealing temperatures. Nonetheless, as highlighted by the red circle in Fig. 3b, a well-defined geometry with edges oriented along high symmetry directions can be occasionally observed. The surface of WS_2_ islands (see Fig. 3b) shows point defects having a bright contrast in STM images (red arrows) and grain boundaries between differently oriented nanocrystals (black arrow).

The growth and morphological characteristics of the inverse heterostructure, *i.e.* MoS_2_ on WS_2_/Au(111), revealed by STM are essentially the same as those of WS_2_/MoS_2_/Au described above. We report in Fig. S4a and c of the ESI† two images of the inverse heterostructure, taken at different coverage of MoS_2_ on WS_2_/Au. The height of MoS_2_ islands is 5.5 ± 0.5 Å (Fig. S4e†), *i.e.* the same as that of WS_2_ on MoS_2_/Au(111), and higher resolution images (Fig. S4b and d†) show a very similar surface morphology.

**Fig. 4:**
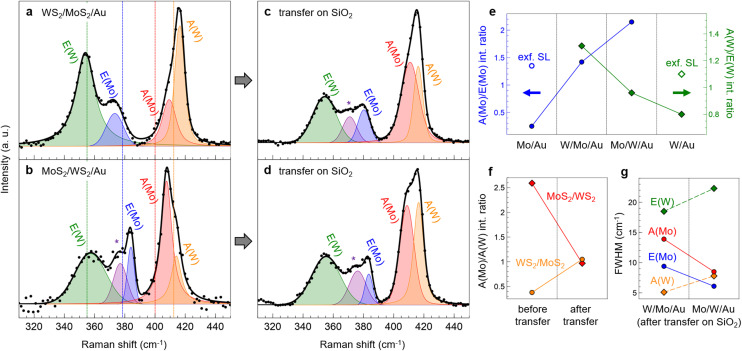
Raman spectra (at 457 nm) of WS_2_/MoS_2_ (a and c) and MoS_2_/WS_2_ (b and d) before (a and b) and after (c and d) transfer on SiO_2_. Legend: black dots = experimental points; solid black line = total fit; colored curves = labeled Voigt functions contributing to the fit. Vertical dotted lines in (a) and (b) mark the positions of the primary Raman peaks of PLD-grown single layers as shown in Fig. 1g and h. (e) A/E intensity ratio of MoS_2_ (blue dots) and WS_2_ (green squares) in single-layer and heterobilayer samples. From left to right: MoS_2_/Au, WS_2_/MoS_2_/Au, MoS_2_/WS_2_/Au, WS_2_/Au. The blank dot and square in the first and last panels refer to the intensity ratio reported for exfoliated SL MoS_2_ and SL WS_2_, respectively.^23^ (f) A(Mo)/A(W) intensity ratio of WS_2_/MoS_2_ (orange circles) and MoS_2_/WS_2_ (red squares) measured before (left) and after (right) the transfer on silica. (g) FWHM of the primary Raman peaks in WS_2_/MoS_2_ (left) and MoS_2_/WS_2_ (right) after the transfer on SiO_2_: E(W) in green, E(Mo) in blue, A(Mo) in red, and A(W) in orange.

In Fig. 4a and b we report the Raman spectra of the two heterostructures, namely WS_2_/MoS_2_/Au (a) and MoS_2_/WS_2_/Au (b). Both spectra show the in-plane and out-of-plane vibrational modes of MoS_2_ and WS_2_ that will be denoted as E(Mo), E(W) and A(Mo), A(W), respectively. In the literature, the Raman spectra of mechanically stacked WS_2_/MoS_2_ heterobilayers are described as “additive”, *i.e.* peaks do not exhibit a shift in position or a change in their relative intensities with respect to the single layers, except for a small upshift of the A mode in the bottom layer.^5^ In our PLD-grown heterostructures, instead, most peaks are affected by the growth procedure, the deposition order of TMD layers, and the influence of the Au surface.

Taking as a reference the peak positions in SL MoS_2_/Au and WS_2_/Au (dashed vertical lines in Fig. 4a and b), we discuss the frequency variations in the vibrational modes of the Au-supported heterobilayers, which are also shown in Fig. S5a (left panel) of the ESI.† In WS_2_/MoS_2_/Au (Fig. 4a), the E(W) peak (green) remains unshifted at 354 cm^−1^, while E(Mo) (blue) downshifts to 373 cm^−1^. The latter is still influenced by the contact with Au(111), as discussed above for SL MoS_2_/Au (Fig. 1g). The high-frequency feature is contributed by A(Mo) (red) at 409 cm^−1^ and A(W) (orange) at 416 cm^−1^; we notice that the A(Mo) peak is not split as was in SL MoS_2_/Au. The hardening of both modes is indicative of a strong vertical coupling typical for increasing thickness to multi-layer configurations with highly pure interfaces, as reported for TMD homobilayers.^23,25,31^

In the inverse heterostructure, *i.e.* MoS_2_/WS_2_/Au (Fig. 4b), we notice a difference in the position of E(Mo) (blue), which is at 384 cm^−1^, and the appearance of an additional peak (purple) at 377 cm^−1^. In literature, the latter is attributed to disorder in the heterobilayer structure, possibly related to defects and W–Mo intermixing.^38–40^ As discussed above, the highly energetic PLD process can indeed increase the disorder in the bottom layer, and the subsequent annealing in UHV can promote W–Mo intermixing, thus it is not currently possible to exclude a certain degree of alloying in the resulting heterostructures. We did not resolve this feature as a separate contribution in Fig. 4a likely because it overlaps with E(Mo), which indeed is wider than in Fig. 4b. The upshift of E(Mo) in the MoS_2_/WS_2_/Au heterostructure is related to the buffer action of the base WS_2_ layer, which shields from the influence of the metallic substrate and restores the peak frequency to values typical of exfoliated samples. As mentioned for SL WS_2_/Au, the position of E(W), here at 357 cm^−1^, is instead less affected by the interaction with Au and it can be considered constant within the experimental error; however, its large width in both heterobilayers suggests that unresolved contributions may be present, *e.g.* the 2LA(M) and TO(M) modes as identified in Fig. 1h. The high-frequency feature in Fig. 4b has a different shape when compared to that in 4a, ascribable to the larger contribution of A(Mo) (red) with respect to A(W) (orange). In MoS_2_/WS_2_/Au, both peaks are slightly downshifted if compared to the inverse heterostructure, respectively at 408 cm^−1^ and 414 cm^−1^; nonetheless, since both modes are hardened with respect to the single layers, an equivalently strong interlayer coupling can be inferred.

The A(Mo)–E(Mo) and A(W)–E(W) frequency differences in both heterobilayers are shown in Fig. S5b (left panel) of the ESI.† The A(W)–E(W) difference is close to the value in WS_2_/Au when WS_2_ is the bottom layer, *i.e.* interfaced with gold, while it shifts towards values typical of free-standing WS_2_ when it is the top layer. The behavior of the A(Mo)–E(Mo) difference is instead less straightforward, especially considering the very high value of 36 cm^−1^ when MoS_2_ is the bottom layer, which could be the result of the interaction with Au combined with the high defectivity caused by the PLD process.

In comparison to the Au-supported single layers (Fig. 1g and h), the intensity of the A modes with respect to the E modes increases in the heterobilayers. The A/E intensity ratio of the two materials is reported in Fig. 4e for the different configurations (for the intensity of the split A(Mo) peak in SL MoS_2_/Au, we chose the more upshifted contribution, as justified above). We observe an increasing trend in which the intensity ratio is minimum in the single-layer configuration and maximum when the layer – whether MoS_2_ or WS_2_ – is on top of the bilayer structure. This behavior results from two counteracting effects: (i) the interfacial interaction with the Au substrate, which damps the out-of-plane vibration in the interfaced TMD layer, and (ii) the vertical coupling with the other TMD layer that enhances the A mode intensity. Indeed, the intermediate case is for the MoS_2_ (WS_2_) layer sandwiched between Au and the WS_2_ (MoS_2_) layer. Therefore, the A/E intensity ratio is very sensitive to interface effects and can be used as a parameter to characterize the heterostructure architecture on strongly interacting substrates.

To explore the possibility of transferring the heterobilayers onto a different substrate, we applied a wet etching transfer procedure (described in Materials and methods and in the ESI, Fig. S1a–e†) aiming at removing the gold substrate and obtaining both heterobilayers supported on silica films. The transfer procedure was indeed successful, after which the samples showed clear contrast by both visual inspection and optical microscopy (Fig. S1f and g†). By performing Raman spectroscopy measurements after the transfer, we got further insight into the interlayer coupling and the Au influence on the vibrational properties. In Fig. 4c and d we show the Raman spectra taken after the transfer, while the differences in peak frequency before and after the transfer are better highlighted in Fig. S5a.†

Comparing Fig. 4a and c, the E(Mo) mode (blue), which we showed to be sensitive to the Au contact, upshifts from 373 to 380 cm^−1^, thus partially restoring the modification induced by Au, approaching the value typical of exfoliated SL MoS_2_. In the inverse heterostructure (Fig. 4d), instead, the position of E(Mo) is not affected by the transfer, since the MoS_2_ layer was not in direct contact with Au. The upshift of E(Mo) after the transfer allows to deconvolve the peak at 372 cm^−1^ (purple) as a separate contribution. As explained above, features in the 372–377 cm^−1^ range can be associated to disorder in the heterostructure.^38–40^ Within the experimental error, no significant differences are observed in both transferred heterobilayer for what concerns the frequency of A(Mo) (red) at 408–410 cm^−1^, while E(W) (green) and A(W) (orange) converge to 355 and 416 cm^−1^, respectively. Significantly, the position of the A(Mo) and A(W) modes is not restored to that of exfoliated SL MoS_2_ and WS_2_ even after the transfer, at variance with what is observed in mechanically stacked heterobilayers.^5^ Out-of-plane modes with similar characteristics have been observed in MoS_2_/WS_2_ heterostructures grown by PLD directly on SiO_2_.^41^ Therefore, we conclude that the enhanced vertical pairing between the TMD layers is not caused by the presence of the Au surface, but rather by the fabrication process. Indeed, the PLD system operates in a UHV environment that guarantees low contamination conditions and thus highly pure interfaces, which is fundamental for a strong interlayer coupling.

The shift in peak frequency after the transfer reflects the A–E frequency difference in MoS_2_ and WS_2_, as shown in the ESI, Fig. S5b† (right panel). In both transferred heterostructures, A(W)–E(W) converges to 62 cm^−1^, which is close to the value of exfoliated WS_2_ homobilayers.^25^ Similarly, A(Mo)–E(Mo) tends to converge towards the value of exfoliated MoS_2_ homobilayers (22 cm^−1^),^24^ but the high defectivity of the bottom MoS_2_ layer and the effect of the interlayer coupling keep the A(Mo)–E(Mo) difference at much higher values.

In the out-of-plane feature, the A(Mo) and A(W) contributions have approximately the same intensity after the transfer, at variance with the Au-supported heterobilayers. We highlight this observation in Fig. 4f, reporting the intensity ratio A(Mo)/A(W) for both WS_2_/MoS_2_/Au (orange dots) and MoS_2_/WS_2_/Au (red squares) heterobilayers before (left) and after (right) the transfer on silica. Evidently, the A(Mo)/A(W) ratio is lower than 1 – *i.e.* A(Mo) is less intense than A(W) – when MoS_2_ is in direct contact with Au, while the opposite is true when the WS_2_ is the interfaced layer. Therefore the interaction with the gold substrate has the effect of damping out-of-plane vibrations only in the bottom layer. As further confirmation, the removal of the Au substrate leads to a more symmetric contribution of A(Mo) and A(W), *i.e.* the A(Mo)/A(W) ratio is about 1 in both heterostructures.

In Fig. 4g we show the full width at half maximum (FWHM) of the primary Raman peaks in WS_2_/MoS_2_ (left) and MoS_2_/WS_2_ (right) after the transfer on silica. We notice that peaks are wider (larger FWHM) when the corresponding TMD layer is on the bottom of the heterostructure, and narrower when on top. This observation further confirms the high defectivity and degree of disorder of the bottom layer, presumably caused by the highly energetic PLD process. The high defect density in the heterostructure is likely also responsible for the lack of photoluminescence (PL) signal in the heterobilayers, whose spectra are shown in Fig. S6† before (b) and after (c) the transfer. In literature, SL MoS_2_ and WS_2_ on SiO_2_ present intense PL around 1.90 and 1.95 eV, respectively,^42,43^ while our PLD-grown single layers on gold (Fig. S6a†) do not exhibit any PL signal, possibly due to charge transfer effects induced by the contact with a highly pure Au surface.^11,44^ However, PL is still absent in the heterobilayers even after the removal of the Au substrate, suggesting that the defects of the TMD layers, and in particular the grain boundaries among the coalesced nanocrystals, can act as non-radiative recombination sites and thus quench the PL signal.

## 4 Conclusions

We developed a PLD procedure to grow WS_2_/MoS_2_ heterobilayers on Au(111) in UHV. *In situ* STM observations allowed us to study the heterostructure morphology depending on the number of laser pulses in the PLD process. Our analysis revealed the possibility of finely tuning the top layer coverage in the monolayer range. We compared two different heterobilayer architectures, having either MoS_2_ or WS_2_ as the base layer interfacing the Au surface. Raman spectroscopy reveals differences in the vibrational properties of the two heterobilayer architectures related to the interaction with the Au substrate, which mostly affects the base layer, in particular the intensity of the out-of-plane mode – either A(Mo) or A(W) – and the position of the in-plane E(Mo) mode. Moreover, Raman analysis suggests a strong interlayer coupling between MoS_2_ and WS_2_, likely the result of the fabrication procedure in UHV that guarantees highly pure interfaces. These results were also confirmed by performing Raman spectroscopy after sample transfer on silica, through a technique that successfully relocated the heterobilayers without weakening the vertical coupling. Our work explores the application of PLD in the synthesis of TMD heterostructures and in relation to specific substrate characteristics, showing potential impact on the future production of novel multi-elemental 2D materials.

## Conflicts of interest

There are no conflicts to declare.

## Supplementary Material

NR-015-D3NR00614J-s001

## References

[cit1] Wang Q. H., Kalantar-Zadeh K., Kis A., Coleman J. N., Strano M. S. (2012). Nat. Nanotechnol..

[cit2] Britnell L., Ribeiro R. M., Eckmann A., Jalil R., Belle B. D., Mishchenko A., Kim Y.-J., Gorbachev R. V., Georgiou T., Morozov S. V. (2013). et al.. Science.

[cit3] Hill H. M., Rigosi A. F., Rim K. T., Flynn G. W., Heinz T. F. (2016). Nano Lett..

[cit4] Pesci F. M., Sokolikova M. S., Grotta C., Sherrell P. C., Reale F., Sharda K., Ni N., Palczynski P., Mattevi C. (2017). ACS Catal..

[cit5] Tongay S., Fan W., Kang J., Park J., Koldemir U., Suh J., Narang D. S., Liu K., Ji J., Li J. (2014). et al.. Nano Lett..

[cit6] Chen Y., Sun M. (2021). Nanoscale.

[cit7] Vishwanath S., Liu X., Rouvimov S., Mende P. C., Azcatl A., McDonnell S., Wallace R. M., Feenstra R. M., Furdyna J. K., Jena D. (2015). et al.. 2D Mater..

[cit8] Mortelmans W., Nalin Mehta A., Balaji Y., Sergeant S., Meng R., Houssa M., De Gendt S., Heyns M., Merckling C. (2020). ACS Appl. Mater. Interfaces.

[cit9] Seo S., Kim S., Choi H., Lee J., Yoon H., Piao G., Park J.-C., Jung Y., Song J., Jeong S. Y. (2019). et al.. Adv. Sci..

[cit10] Pielić B., Novko D., Rakić I. v., Cai J., Petrović M., Ohmann R., Vujičić N., Basletić M., Busse C., Kralj M. (2021). ACS Appl. Mater. Interfaces.

[cit11] Tumino F., Casari C. S., Passoni M., Russo V., Li Bassi A. (2019). Nanoscale Adv..

[cit12] Tumino F., Grazianetti C., Martella C., Ruggeri M., Russo V., Li Bassi A., Molle A., Casari C. S. (2021). J. Phys. Chem. C.

[cit13] Loh T. A. J., Chua D. H. C. (2014). ACS Appl. Mater. Interfaces.

[cit14] Loh T. A. J., Chua D. H. C., Wee A. T. S. (2015). Sci. Rep..

[cit15] Serna M. I., Yoo S. H., Moreno S., Xi Y., Oviedo J. P., Choi H., Alshareef H. N., Kim M. J., Minary-Jolandan M., Quevedo-Lopez M. A. (2016). ACS Nano.

[cit16] Yao J.-D., Zheng Z.-Q., Yang G.-W. (2019). Prog. Mater. Sci..

[cit17] Sørensen S. G., Füchtbauer H. G., Tuxen A. K., Walton A. S., Lauritsen J. V. (2014). ACS Nano.

[cit18] Velicky M., Donnelly G. E., Hendren W. R., McFarland S., Scullion D., DeBenedetti W. J., Correa G. C., Han Y., Wain A. J., Hines M. A. (2018). et al.. ACS Nano.

[cit19] Yang P., Zhang S., Pan S., Tang B., Liang Y., Zhao X., Zhang Z., Shi J., Huan Y., Shi Y. (2020). et al.. ACS Nano.

[cit20] Velickỳ M., Donnelly G. E., Hendren W. R., DeBenedetti W. J., Hines M. A., Novoselov K. S., Abruña H. D., Huang F., Frank O. (2020). Adv. Mater. Interfaces.

[cit21] Chakraborty B., Matte H. S. S. R., Sood A. K., Rao C. N. R. (2013). J. Raman Spectrosc..

[cit22] Nečas D., Klapetek P. (2012). Open Phys..

[cit23] Zhang X., Qiao X.-F., Shi W., Wu J.-B., Jiang D.-S., Tan P.-H. (2015). Chem. Soc. Rev..

[cit24] Li H., Zhang Q., Yap C. C. R., Tay B. K., Edwin T. H. T., Olivier A., Baillargeat D. (2012). Adv. Funct. Mater..

[cit25] Zhao W., Ghorannevis Z., Amara K. K., Pang J. R., Toh M., Zhang X., Kloc C., Tan P. H., Eda G. (2013). Nanoscale.

[cit26] Grønborg S. S., Ulstrup S., Bianchi M., Dendzik M., Sanders C. E., Lauritsen J. V., Hofmann P., Miwa J. A. (2015). Langmuir.

[cit27] Krane N., Lotze C., Franke K. J. (2018). Surf. Sci..

[cit28] Wakabayashi N., Smith H., Nicklow R. (1975). Phys. Rev. B: Solid State.

[cit29] Schutte W., De Boer J., Jellinek F. (1987). J. Solid State Chem..

[cit30] Tumino F., Casari C. S., Li Bassi A., Tosoni S. (2020). J. Phys. Chem. C.

[cit31] Lee C., Yan H., Brus L. E., Heinz T. F., Hone J., Ryu S. (2010). ACS Nano.

[cit32] Yasuda S., Takahashi R., Osaka R., Kumagai R., Miyata Y., Okada S., Hayamizu Y., Murakoshi K. (2017). Small.

[cit33] Pollmann E., Sleziona S., Foller T., Hagemann U., Gorynski C., Petri O., Madauß L., Breuer L., Schleberger M. (2021). ACS Omega.

[cit34] Shi W., Lin M.-L., Tan Q.-H., Qiao X.-F., Zhang J., Tan P.-H. (2016). 2D Mater..

[cit35] Yang J., Lee J.-U., Cheong H. (2017). FlatChem.

[cit36] Rong Z. Y., Kuiper P. (1993). Phys. Rev. B: Condens. Matter Mater. Phys..

[cit37] Diaz H. C., Chaghi R., Ma Y., Batzill M. (2015). 2D Mater..

[cit38] Mignuzzi S., Pollard A. J., Bonini N., Brennan B., Gilmore I. S., Pimenta M. A., Richards D., Roy D. (2015). Phys. Rev. B: Condens. Matter Mater. Phys..

[cit39] Yao J., Zheng Z., Yang G. (2016). ACS Appl. Mater. Interfaces.

[cit40] Chen Y., Dumcenco D. O., Zhu Y., Zhang X., Mao N., Feng Q., Zhang M., Zhang J., Tan P.-H., Huang Y.-S. (2014). et al.. Nanoscale.

[cit41] Sinha S., Kumar S., Arora S. K., Sharma A., Tomar M., Wu H.-C., Gupta V. (2021). J. Appl. Phys..

[cit42] Mak K. F., Lee C., Hone J., Shan J., Heinz T. F. (2010). Phys. Rev. Lett..

[cit43] Gutiérrez H. R., Perea-López N., Elías A. L., Berkdemir A., Wang B., Lv R., López-Urías F., Crespi V. H., Terrones H., Terrones M. (2013). Nano Lett..

[cit44] Shi J., Ma D., Han G.-F., Zhang Y., Ji Q., Gao T., Sun J., Song X., Li C., Zhang Y. (2014). et al.. ACS Nano.

