# Supplementary material for: Interface coupling in Au-supported MoS2–WS2 heterobilayers grown by pulsed laser deposition
Source: Nanoscale. 2023 Mar 31;15(16):7493–501. doi: 10.1039/d3nr00614j (PMC10134180; doi:10.1039/d3nr00614j)
Supplement: NR-015-D3NR00614J-s001 [file NR-015-D3NR00614J-s001.pdf]

Electronic Supplementary Material (ESI) for Nanoscale.
This journal is © The Royal Society of Chemistry 2023

SUPPLEMENTARY INFORMATION

Interface coupling in Au-supported MoS_2–WS_2 heterobilayers grown by pulsed laser deposition

Paolo D'Agosta*†, Francesco Tumino*†, Valeria Russo*, Andrea Li Bassi*, Carlo S. Casari*

* Department of Energy, Politecnico di Milano, via G. Ponzio 34/3, I-20133 Milan, Italy

† Email: paolo.dagosta@polimi.it, francesco.tumino@polimi.it

Transfer on silica

The Au-supported WS_2/MoS_2 and MoS_2/WS_2 heterobilayers were successfully transferred on 300 nm thick SiO_2 films thermally grown on Si. The transfer process is summarized in figure S1.

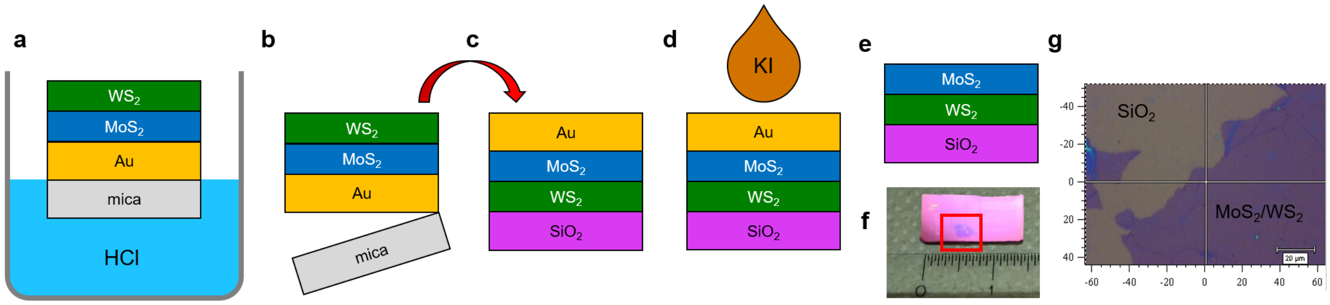

Figure S1: Schematic of the transfer procedure. (a) The sample is set afloat in a 37% (w/w) HCl solution that etches the Au-mica interface. (b) The mica substrate is mechanically removed and (c) the Au-supported heterostructure is placed on a silica substrate with the Au substrate facing up. (d) A few drops of a potassium monoiodide solution are dropped on the sample surface to etch away the Au film. (e) The sample is rinsed in deionized water and isopropanol and left to dry in air. (f) Photograph of the heterobilayer transferred on SiO_2, recognizable as the darker spot on the surface inside the red square. (g) Image of the heterobilayer sample (purple) acquired by optical microscopy after the transfer on silica (grey).

Single layer MoS_2 on Au(111)

Higher resolution images of SL MoS_2 on Au(111) are shown in figure S2, highlighting in particular (a) the sharp, polygonal edges of the coalescing nanocrystals, (b) the moiré and atomic periodicity, and (c) the apparent height of the layer on the gold surface.

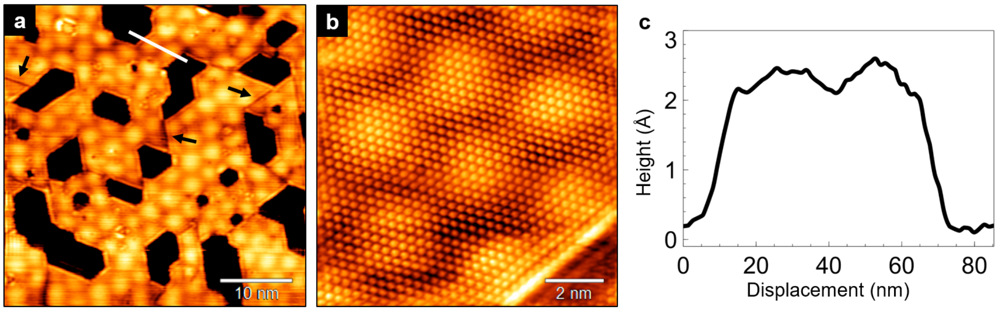

Figure S2: High resolution STM images of SL MoS_2/Au(111). (a) Sharp grain boundaries (pointed at by the black arrows) and polygonal holes in the layer indicate the coalescence of differently oriented nanocrystals (1.0 V, 0.5 nA). (b) Atomically resolved image showing the moiré superstructure (0.5 V, 0.3 nA). (c) Topographic profile along the white line in (a).

Homobilayer MoS_2 on Au(111)

In figure S3, STM images of homobilayer MoS_2 on grown Au(111) by PLD are shown. In particular, in the first stages of growth (total coverage of 1.15 ML) we observe a disordered pattern in the underlying layer, which may be attributed to an increased defectivity or to electronic interference effects from top-layer islands.

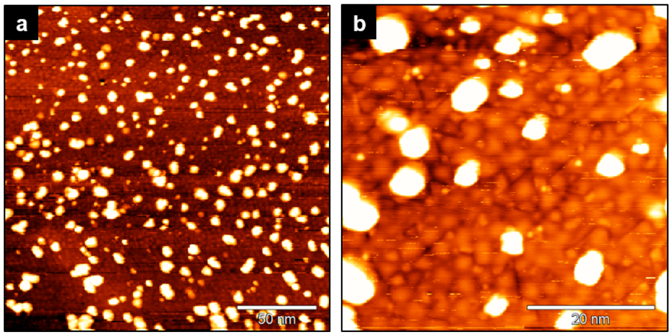

Figure S3: STM images at different magnifications of a homobilayer MoS_2/Au(111) sample, with a coverage of about 1.15 ML. (a) 2.0 V, 0.4 nA. (b) 1.5 V, 0.4 nA. The underlying layer exhibits a disordered pattern.

Inverse heterostructure: MoS_2/WS_2/Au(111)

The growth pattern and morphology of the inverse heterostructure, i.e. MoS_2 grown on SL WS_2/Au(111), are basically the same as those described in-depth for the WS_2/MoS_2/Au(111) heterobilayer. In figure S4, we report selected STM images of MoS_2/WS_2/Au(111) after the first (a,b) and last (c,d) deposition cycles of the top layer.

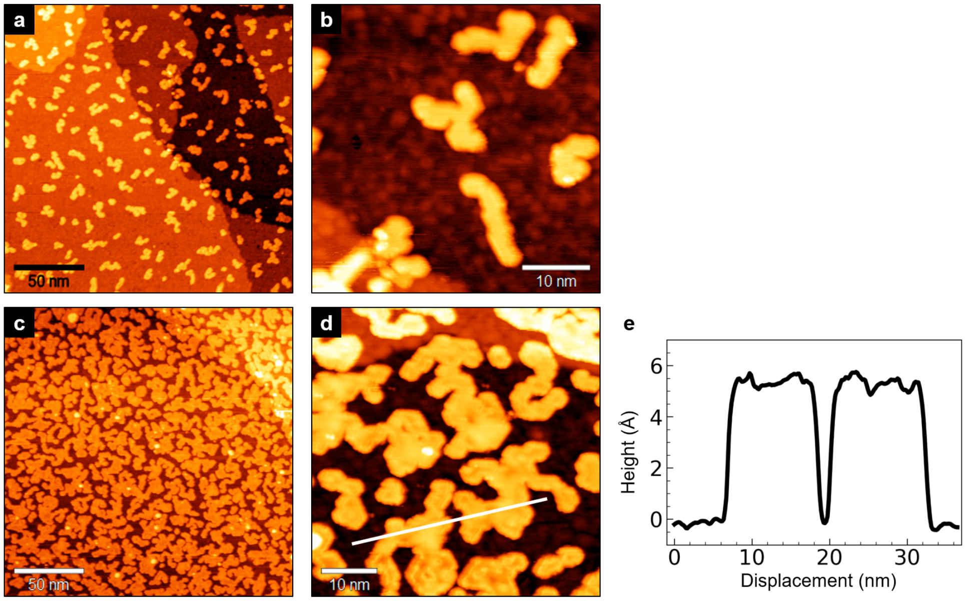

Figure S4: STM images at low (a,b) and high (c,d) coverage of MoS_2 on WS_2/Au(111). The MoS_2 coverage is expressed in fractions of monolayer (ML). (a) 0.19 ML (1.3 V, 0.8 nA). (b) Higher resolution image of the sample in (a) (−1.6 V, 0.4 nA). (c) 0.71 ML (1.3 V, 0.6 nA). (d) Higher resolution image of the sample in (c) (−1.1 V, 0.5 nA). (e) Topographic profile along the white line in (d).

Frequency trends of the primary Raman peaks

In figure S5a, the frequencies (in cm^{-1}) of the out-of-plane (A) and in-plane (E) modes of MoS_2 and WS_2 are reported for the four different heterobilayers configurations, i.e. WS_2/MoS_2 and MoS_2/WS_2 as-grown on the Au substrate and after the transfer on SiO_2. Similarly, in figure S5b the A−E frequency difference is shown for the same four configurations.

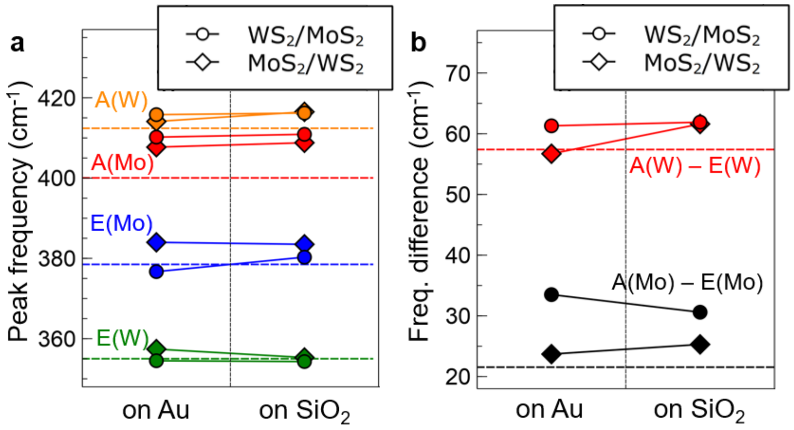

Figure S5: (a) Frequency of the primary Raman peaks in WS_2/MoS_2 (circles) and MoS_2/WS_2 (squares) heterobilayers on the Au substrate (left panel) and after the transfer on SiO_2 (right panel): for MoS_2, E(Mo) is in blue and A(Mo) is in red; for WS_2, E(W) is in green while A(W) is in orange. The dashed colored lines indicate the peak frequency of the same peaks (associated by color) in the Au-supported single layer configuration. (b) Frequency difference between the out-of-plane and the in-plane modes in MoS_2 (i.e. A(Mo)−E(Mo), in black) and in WS_2 (i.e. A(W)−E(W), in red). The value is measured in both WS_2/MoS_2 (circles) and MoS_2/WS_2 (squares) heterostructures on the Au substrate (left panel) and after the transfer on SiO_2 (right panel). The dashed colored lines indicate the A−E frequency difference in SL MoS_2/Au (black) and SL WS_2/Au (red).

Photoluminescence spectra

Photoluminescence (PL) experiments were carried out on the TMD single layers, and on both heterobilayers before and after the transfer on silica; the resulting spectra are shown in figure S6. As is evident by comparing the PL spectra of the samples (red and blue lines) with the spectra collected on the clean substrates (grey lines), we report the absence of any excitonic contribution at all stages of sample fabrication and transfer.

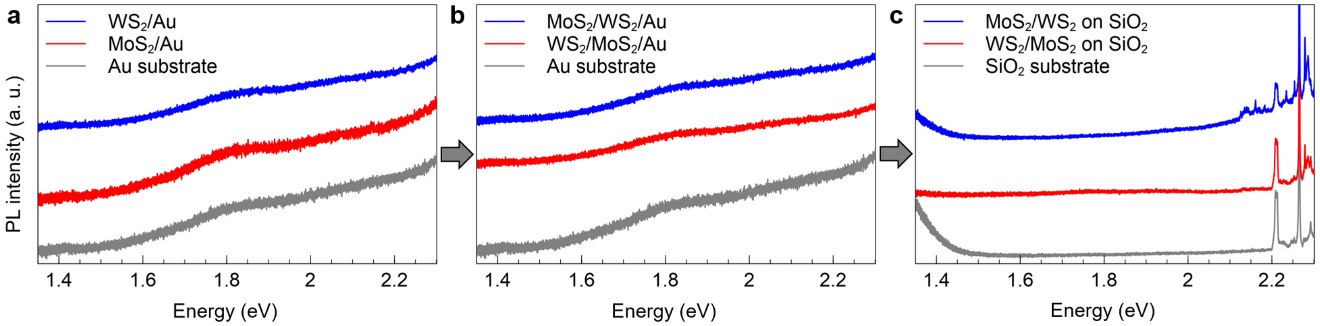

Figure S6: Photoluminescence spectra of (a) the single layers on gold (at 514 nm), (b) the heterobilayers on gold (at 514 nm) and (c) the heterobilayers transferred on silica (at 532 nm). The grey spectra are collected on the gold (a,b) and silica (c) substrates. The red spectra are collected on SL MoS_2 (a) and on WS_2/MoS_2 before (b) and after (c) the transfer on SiO_2. Analogously, the blue spectra are collected on SL WS_2 (a) and on MoS_2/WS_2 before (b) and after (c) the transfer on SiO_2.
